# How Industrial Transfer Processes Impact on Haze Pollution in China: An Analysis from the Perspective of Spatial Effects

**DOI:** 10.3390/ijerph16030423

**Published:** 2019-02-01

**Authors:** Yajie Liu, Feng Dong

**Affiliations:** School of Management, China University of Mining and Technology, Xuzhou 221116, China; tb18070005b2@cumt.edu.cn

**Keywords:** industrial transfer, environment pollution, spatial panel model

## Abstract

Industrial transfer from advanced regions is a good way to foment economic development in less advanced regions. Nevertheless, does industrial transfer intensify or alleviate haze pollution? To answer this question, this study employed the shift-share method and spatial panel models to explore how industrial transfer processes impact haze pollution in the case of China. The main results are as follows: (1) With the advances made in industrial transfer and upgrading, China has entered the stage of decoupling between the economic development level and haze pollution. (2) Industrial transfer could effectively alleviate the degree of haze pollution in the transferred-out areas, but it would have a significant accelerating effect on haze pollution in the transferred-in areas. Compared with non-polluting industries, polluting industries would be responsible for a large deterioration in the local air quality. (3) Environmental regulations, as the main factor mitigating environmental pollution, do not achieve the desired effects and significantly reduce the regional pollution levels that led to haze. Therefore, the effects of industrial transfer should also be comprehensively considered in government of undertake regions. There would likely be great economic costs if the old path of “pollution first and treatment later” is followed. This study not only advances the existing literature, but also is of considerable interest to policy makers.

## 1. Introduction

The coexistence of industrialization and urbanization in China has caused increasingly serious haze pollution [1,2,3]. The main haze components are inhalable particulate matter and fine particles, which can pose a serious threat to human health, and their continued industrial emission will hinder the development of a green and healthy economy in China [4]. Data published by the World Health Organization (WHO), has revealed that about seven million people worldwide die from conditions related to fine particulate matter in polluted air every year, with around two million of these deaths occurring in China due to exposure in either outdoor or indoor air [5]. Taking the 20 provinces and cities affected by haze pollution in January 2013 as an example, the direct economic losses to transportation and health reached RMB 23 billion [6]. In 2015, Beijing’s PM_2.5_ concentration reached 1000 μg⁄m^3^, 100 times the level of the haze standard set by the WHO [7,8]. Due to the wide range of areas affected by haze pollution, the hidden dangers to people’s health, and the huge losses caused to the economy [9], the Chinese government has made a great effort to treat atmospheric pollution and has launched a “blue sky” protection campaign to control air pollution in key regions [10,11,12]. The State Council promulgated the “Action Plan on Air Pollution Prevention and Control” (hereinafter referred to as “Ten Articles of Atmosphere”) in September 2013. Ten measures for air pollution prevention and control were proposed, in which Article 5 involved strict energy conservation and environmental protection measures, optimization of the layout of industrial space, strengthening of environmental supervision and the prohibition of the transfer of outdated production methods [13].

To optimize industrial layout, in 2012 the Ministry of Industry and Information Technology released the “Guidance Catalogue on Industrial Transfer,” which established an industrial development pattern featuring “one axis, one belt, five circles and five groups.” It also involved the industries in the coastal areas shifting to the central and western regions, with the central region also adopting staged development strategies to actively undertake industrial transfer from the eastern region. As a strategic decision in China’s industrial policy, industrial transfer not only promotes the transformation and upgrading of China’s industry, but also is an important practice to optimize the spatial layout of industry [14]. Some industrial transfer achievements have already been made [15], and the spatial layout of industry has gradually become clear in the Beijing-Tianjin-Hebei region, which not only specifically explains the non-capital function of Beijing, but also has played an important role in driving industrial transformation and the upgrading of neighboring provinces and cities. By focusing on electronic information, precision equipment, automobiles, household appliances, and clothing and textiles, the Yangtze River Economic Belt has undergone industrial transfer and clustering to develop an industrial spatial layout that is compatible with the carrying capacity of resources and the environment. In 2016, the industrial added-value of the Yangtze River Economic Belt was RMB 97,835.33 billion and regional production totaled RMB 259,941.66 billion, increasing by 59.87 and 85.5% (calculated at flexible prices), respectively, compared to 2010 values. Industrial transfer has led to rapid economic development in adjacent areas, the upgrading of industrial structure in the receiving area, and the optimization of industrial spatial layout. However, it is still unknown whether the spatial optimization of industrial areas brought about by industrial transfer can alleviate haze pollution.

Most studies of the effect of regional industrial transfer have focused on economic effects or the effects of technology spillover [16,17], while the environmental effects of regional industrial transfer remain controversial. For example, industrial transfer in the Beijing-Tianjin-Hebei region does not exacerbate environmental pollution in the region [18], while industrial transfer in the central region is accompanied by pollution leakage [19]. Industrial transfer has been extended from the pilot areas to the national level; however, studies of the regional environmental effects not only lack a consideration of the overall process, have also not accurately measured the environmental impacts of industrial transfer on the receiving areas. To objectively evaluate the impact of regional industrial transfer on haze pollution in China, and propose an economic method to control haze pollution from the perspective of industrial transfer, 30 provinces in China from 2008 to 2016 were used as samples. The study adopted the spatial panel model to analyze the haze pollution effects of industrial transfer based on the spatial spillover of haze pollution. 

The remainder of the paper is organized as follows: Section 2 reviews the previous related studies. The research methods and data are described in Section 3. The empirical results are presented and discussed in Section 4. Finally, we conclude our study in Section 5.

## 2. Literature Review

Both international and regional industrial transfer can generate adverse environmental effects and have been considered in previous studies. The status of international industrial transfer is usually measured in terms of foreign direct investment (FDI), but a consensus on the environmental effects of FDI is yet to be reached. The pollution haven and pollution halo hypotheses have been proposed. Copeland and Taylor [20] first proposed the pollution haven hypothesis based on a study of the relationship between northern and southern trade and the environment. According to the hypothesis, the free flow of production factors encourages pollution-intensive industries to use FDI as a carrier to transfer to countries with loose environmental regulations, and areas with lower environmental regulation standards will then become a haven for polluting industries [21,22]. Subsequently, many Chinese and international researchers have incorporated control variables such as economic growth, R&D level, energy consumption, and urbanization into their research to verify the pollution haven hypothesis and estimate the impact of FDI on environmental pollution. However, some researchers hold the opposite view and believe that FDI improves the environmental quality of host countries through economic growth and the introduction of high level technologies. For example, when Liang [23] explored the relationship between urban air pollution, industrial structure, and FDI in China, a significant negative correlation between FDI and air pollution was discovered, with FDI deemed to be beneficial to the environment of the host country. Deng and Xu [24] used a spatial lag model (SLM) and a spatial error model (SEM) to analyze the relationship between FDI and environmental pollution in various provinces in China during 2000–2009, and concluded that the pollution haven hypothesis was not established, with FDI significantly easing China’s environmental pollution. In some studies, the two viewpoints expressed above have been combined. For example, Liu et al. [25] used an SLM and SEM to analyze the impact of China’s FDI inflow on different types of environmental pollution and discovered that the impact of FDI was significantly different for different types of environmental pollution. This confirmed the existence of both the pollution haven and pollution halo hypotheses.

There are fewer studies of the impact of regional industrial transfer on the environment than the environmental effects of international industrial transfer. Zheng et al. [26] analyzed the water pollution status in industrial export areas and areas undertaking various industrial activities, and discovered that industrial transfer of electronics, plastics and bio-pharmaceutics caused the transfer of water pollution. Lin and Zou [27] employed the ACT model to test the environmental effects of China’s regional industrial transfer based on data from 2000 to 2011, and found that industrial transfer between the east and the west has accelerated the transfer of pollution. Zhang and Gou [28] analyzed Chinese provincial data and found that the transfer of polluting industries has accelerated the pace of environmental pollution in underdeveloped areas; however, it was concluded that targeted environmental regulations would generate a win-win result for the environment and the economy. Dou and Shen [19] explored the environmental impact of pollution-intensive industries in central China, and found that the transfer of polluting industry has led to the transfer of pollutants such as wastewater, SO_2_ and soot to the central region. Xu et al. [29] studied the relationship between regional industrial transfer and carbon transfer in China, and found that the relationship between industrial transfer and carbon transfer had an “inverted U” shape. Although not all provinces had exceeded the turning point, industrial transfer had increased the transfer of carbon emissions.

There are two main categories of research on haze pollution, i.e., studies of the components and major influencing factors of haze, and studies of solutions to haze pollution. The main components of haze are SO_2_, NO_2_, and inhalable particulate matter, in which the inhalable particles when combined with fog will become haze. Natural factors are only part of the cause of haze pollution, while socio-economic factors are the root cause of serious haze pollution in China. Haze is mainly a consequence of the lack of development of new energy technologies, with coal remaining as the main energy source, a high proportion of heavy industry in the industrial mix, motor vehicles as the main means of transportation and a large amount of dust generated by construction sites as urbanization proceeds. Further studies have shown that factors such as fiscal decentralization, energy price distortion, and environmental regulation are positively related to haze pollution, while industrial clustering will effectively alleviate the degree of haze pollution. The government is currently mainly focused on economic solutions to vigorously control haze, including taxation policies based on a resource tax [30], sulfur tax [31], and carbon tax [32,33]. Other countermeasures are also proposed, such as improving the system of atmospheric emission rights trading, perfecting the laws and regulations in place for haze management, and building a regional haze joint prevention and control mechanism.

Some studies have suggested that industrial transfer will have an impact on the status of haze pollution in each province. Leng et al. [34] analyzed data for China’s provinces and confirmed that FDI is positively related to haze pollution in China; however, Li et al. [35] undertook a case study of the Pearl River Delta and showed that FDI can effectively alleviate the severity of haze pollution in the region. The study was conducted from the perspective of international industrial transfer, while research on the impact of regional industrial transfer on haze pollution is still limited. Industries in China’s developed regions are moving to underdeveloped regions in an orderly manner. To prevent the underdeveloped regions from continuing to adhere to the old policy of “pollution first and treatment later”, this study explored the relationship between regional industrial transfer and haze pollution in China. It can provide suggestions for the management of haze from the perspective of industrial transfer.

There are defects in existing studies of the effect of national industrial transfer on haze pollution, and there is a need to accurately evaluate the impact of regional industrial transfer on haze pollution. Apart from previous research, this study extends them from the following perspectives: (1) Compared with the research on the issue of international industrial transfer, there are few studies on the environmental pollution caused by industrial transfer at provincial level. It is urgent to study the impact of domestic industrial transfer on haze pollution, especially at the time that the main way to alleviate haze pollution in China is industrial transfer. (2) Based on the deviation-share method and entropy weight method, the industrial transfer index was constructed, which not only excludes the influence of time factor and technological progress, but also takes the policy factor into consideration. (3) We examined the impacts of industrial transfer on haze pollution from the perspectives of level and scale respectively. Moreover, industrial areas were distinguished as undertaking zones and transferring zones. (4) We divided the secondary industry into polluting industry and non-polluting industry, and explored the impact of different industrial types on haze pollution. It will provide relevant suggestions for the central and western regions to undertake proper transferring industry.

## 3. Methodology and Data

### 3.1. Methodology

Cross-regional industrial transfer can result in macro-level industrial restructuring and changes of regional industrial distribution, and its impact on environmental quality should be paid attention to seriously. This is particularly the case for the increasingly serious incidences of air pollution in China, with haze pollution posing serious risks to public health and causing huge economic losses. As industries move among provinces, industrial transfer from a certain province may affect the haze pollution of others. In this study, the impact of industrial transfer on the haze pollution in each province was considered, but it was recognized that ignoring the spatial effects associated with would cause the model to be set incorrectly. Therefore, a spatial econometric model was used to investigate the relationship between industrial transfer and haze pollution, and to measure the spatial spillover effects of various influencing factors.

As spatial econometric analysis technology develops, the most widely used models have changed from spatial auto-regression (SAR) model and spatial error model (SEM) to spatial interaction models (i.e., the spatial Dubin model: SDM) and spatial auto-correlation (SAC) models. The transmission mechanisms and economic implications vary among the different types of models. Containing only the dependent variable lag term, the SAR models assume that the dependent variable will affect haze pollution in other areas through spatial interaction [36]. SEM only contain the spatial auto-correlative error term, with the spatial spillover effect considered a random impact, enabling errors to be transmitted as a result. Both the SDM and the SAC model contain a dependent variable lag term and a spatial auto-correlative error term, with the two spatial effect conduction mechanisms also taken into consideration. In addition, the SDM includes a spatial interaction, meaning that the haze pollution in a certain province is not only affected by a series of local independent variables, but also by the haze pollution of other provinces and its independent variables [37]. Because the setting of a spatial econometric model is key to revealing the correlation between variables, for a better model fitting, this study used the ordinary least squares (OLS)-[SAR\SEM]-SAC-SDM concept in modeling and the Lagrange multiplier statistic, Wald statistic, and likelihood ratio (LR) to test the effectiveness of model fitting. Due to the existence of a spatial lag dependent variable and lag error variable, it could not be assumed that the strict exogenous and residual perturbation terms of the explanatory variables are independent and identically distributed as in a traditional econometric model. 

The spatial dependence of economic variables is taken into account in the construction of a spatial econometric model, and it could be compared with a traditional econometric model. It could not be assumed that the strict exogenous and residual disturbance terms of the explanatory variable in the traditional measurement model are independent and identically distributed because of the existence of a spatial lag variable and lag error variable. Methods such as the instrumental variable method [38,39,40,41,42] or maximum likelihood estimation method [43,44,45,46] need to be selected for model estimation. Because it is very difficult for the instrumental variables (IV) method to select suitable variable tools, and its parameter estimation results tend to exceed the domain, a maximum likelihood estimation (MLE) was employed to estimate the model and avoid the problems described above. 

The following models were constructed based on the information given above:

(1) $$LnY_{it}=\beta_{0}+\delta WLnY_{it}+\beta_{1}LnX_{it}+\beta_{2}LnX_{control}+\theta_{1}WLnX_{it}+\theta_{2}WLnX_{control}+\varepsilon_{it}$$

(2) $$\begin{array}{l} LnY_{it}=\beta_{0}+\delta WLnY_{it}+\beta_{1}LnX_{it}+\beta_{2}LnX_{control}+\mu_{it}\\ \mu_{it}=\lambda W\mu_{it}+\varepsilon_{it} \end{array}$$

Equation (1) is the SDM, while Equation (2) is a SAC model. Partial constraints were added to these models before obtaining SAR, SEM, and OLS models. 

If the spatial interactions examined in the SDM do not exist, the SDM is transformed into an SAC model. In addition, when the spatial auto-correlative error term coefficient in the spatial crossover model is zero, the model is transformed into the SAR model, e.g., Equation (3):

(3) $$LnY_{it}=\beta_{0}+\delta WLnY_{it}+\beta_{1}LnX_{it}+\beta_{2}LnX_{control}+\varepsilon_{it}$$

When $\theta_{i}+\delta \beta_{i}=0$ is obtained in the SDM, or when the spatial lag term coefficient in the SAC model is zero, the model is transformed into an SEM, e.g., Equation (4):

(4) $$\begin{array}{l} LnY_{it}=\beta_{0}+\beta_{1}LnX_{it}+\beta_{2}LnX_{control}+\mu_{it}\\ \mu_{it}=\lambda W\mu_{it}+\varepsilon_{it} \end{array}$$

When spatial correlation is not considered in the model, it is transformed into an OLS model, with all spatial correlation coefficients being zero, e.g., Equation (5):

(5) $$LnY_{it}=\beta_{0}+\beta_{1}LnX_{it}+\beta_{2}LnX_{control}+\varepsilon_{it}$$

Here, *Y_it_* is the annual average PM_10_ concentration in the provinces in year t; *X_it_* is the core explanatory variable-industry transfer, and the detailed measurement method is as follows. The industrial transfer status of each province was measured from multiple dimensions, and the corresponding model was built for an analysis of the industrial transfer level (*IT*), non-polluting industry transfer level (*IT_N_*), polluting industry transfer level (*IT_P_*), the scale of industrial transfer (*SIT*), the scale of non-polluting industry transfer (*SIT_N_*), and the scale of polluting industry transfer (*SIT_P_*). *X_control_* refers to a series of control variables, including population density, GDP per capita, street lighting brightness, energy mix, vehicle use, environmental regulations, industrial structure, rainfall and humidity. *μ_it_* and *ε_it_* are random disturbances subject to a normal distribution; W is a spatial weighting matrix, with elements generally determined in terms of the spatial unit’s connectivity. If the regions are adjacent the elements value is one, otherwise the value is zero. Because the relationship between the regions cannot be fully reflected in the geographic adjacency matrix, and the haze spillover effect is related to the distance between the two provinces, with a larger distance between provinces, the spatial spillover effect of haze will be weakened. Therefore, the spatial weighting matrix was constructed based on the gravity model:

(6) $$w_{ij}=\{\begin{array}{c} \frac{\bar{PM_{i}}*\bar{PM_{j}}}{d_{ij}}\;,i\neq j\\ 0\;\;\;\;\;\;\;,i=j \end{array}$$

Here, *i* and *j* represent each province; $\bar{PM_{i}}$ is the average PM_10_ concentration in province *i* from 2008 to 2016; and *d_ij_* is the linear distance to the geographical center of the province (km).

### 3.2. Variables and Data

#### 3.2.1. Interpreted Variable

*PM_10_ concentration*. PM_2.5_ data has only been available in China since the end of 2012, and most of the literature refers to the global average annual PM_2.5_ concentration published by the Center for Social and Economic Data and Applications of Columbia University. The data covers the period from 1998 to 2012; however, the data are found to be of poor quality, with the only usable data covering the period from 1999 to 2011. The focus of this study is the spatial spillover effect of industrial transfer on haze pollution, but the available data do not cover the critical period of China’s industrial transfer. There are gaps in the urban PM_2.5_ concentration data published by China’s environmental protection departments for different major cities. Particulates are an important constituent of haze, but due to the gaps in PM_2.5_ statistical data and the wide availability of PM_10_ concentration data, the PM_10_ concentration in provincial capitals (unit: μg/m^3^) was used as an indicator of the haze level in each province [47].

#### 3.2.2. Explanatory Variables

*Industrial transfer*. The characteristics of industrial development influence the industrial transfer process. The location of agriculture, extractive industries, and various service enterprises are related to natural resource endowment and long-distance transportation. Industrial transfer occurs mostly in industrial manufacturing, and the development of manufacturing enterprises can have a significant impact on environmental pollution [48]. Therefore, this study focused on the impact of industrial manufacturing transfer on haze governance in China. Industrial classification refers to the Industrial Classification of the National Economy standard. Because of the differences in the statistical yearbooks and the overall lack of data, the industries were integrated and divided into 20 categories to ensure data consistency and availability. The effect of industrial transfer on haze was decomposed to distinguish between the transfer of polluting and non-polluting industries. Table 1 shows the details of this division.

Based on the shift-share method, changes in the economic output of an industry over a certain period of time can be decomposed into growth components at different regional levels to observe the evolution of spatial and temporal trends, and the absolute scale of inter-regional industrial transfer:

(7) $$Q_{i,t}^{k}-Q_{i,t-1}^{k}=Q_{i,t-1}^{k}*\left(\frac{Q_{C,t}^{k}}{Q_{C,t-1}^{k}}-1\right)+Q_{i,t-1}^{k}*\left(\frac{Q_{i,t}^{k}}{Q_{i,t-1}^{k}}-\frac{Q_{C,t}^{k}}{Q_{C,t-1}^{k}}\right)$$

$Q_{i,t}^{k}$ and $Q_{i,t-1}^{k}$ refer to the total production value of time *t* and *t-1* for industry *k* of province *i*, and $Q_{i,t-1}^{k}*\left(\frac{Q_{C,t}^{k}}{Q_{C,t-1}^{k}}-1\right)$ is the national growth component of the industrial scale. An increase in province *i*, according to the national growth rate of industry *k*, $Q_{i,t-1}^{k}*\left(\frac{Q_{i,t}^{k}}{Q_{i,t-1}^{k}}-\frac{Q_{C,t}^{k}}{Q_{C,t-1}^{k}}\right)$ indicates the province’s growth component of the industrial scale. If the difference between the growth rate of province *i* and the national growth rate is greater than zero, the province experiences an industrial transfer; otherwise there is an industry transferred out of the province.

To obtain comprehensive indicators of industrial transfer in each province, the weighting of potential indicators was determined based on the principle of information entropy of data from the transfers of 20 industries in each province. The following steps were used to process the data to compare the comprehensive indicators of industrial transfer in different provinces for different years.

First, the indicator was selected. Assuming there are *z* years, *x* provinces, and *y* industries transferred, *x_tij_* indicates the transfer value of industry *j* in province *i* in year *t*.

Second, the indicators were standardized. The original indicators were standardized by the following formula to eliminate the influence of the dimension and unit difference between the indicators on the results:

(8) $$\mathrm{Positive}\;\mathrm{indicator}:\;x_{tij}^{\prime }=\frac{x_{tij}-x_{j}^{min}}{x_{j}^{max}-x_{j}^{min}}$$

(9) $$\mathrm{Negative}\;\mathrm{indicator}:\;x_{tij}^{\prime }=\frac{x_{j}^{max}-x_{tij}}{x_{j}^{max}-x_{j}^{min}}$$

where, $x_{j}^{max}$, $x_{j}^{min}$ represent the maximum and minimum values, respectively, in the transfer of industry *j*.

Third, the weighting of the indicator was determined by the principle of information entropy:

(10) $$p_{tij}=\frac{x_{tij}^{\prime }}{\sum_{t}\sum_{i}x_{tij}^{\prime }}$$

Fourth, the entropy value of the jth indicator was calculated:

(11) $$e_{j}=-k\underset{t}{\sum }\underset{i}{\sum }p_{tij}*\mathrm{Ln}\left(p_{tij}\right)$$

where k=ln(z×x).

Fifth, the information utility of indicator j was calculated:

(12) $$g_{j}=1-e_{j}$$

Sixth, the weightings of each indicator were calculated:

(13) $$w_{j}=\frac{g_{j}}{\sum_{j}g_{j}}$$

Finally, the level and scale of industrial transfer in each province were calculated. Using the weighted coefficient *w_j_*, the weighted summation of each industry transfer index was calculated, with the result representing the level and scale of industrial transfer in each province:

(14) $$v_{ti}=\underset{j}{\sum }w_{j}x_{tij}^{\prime }$$

(15) $$V_{ti}=\underset{j}{\sum }w_{j}x_{tij}$$

Based on the above, the variables *IT*, *IT_N_* and *IT_P_* were obtained to measure the industrial transfer level of each province, while the variables *SIT SIT_N_*, and *SIT_P_* were obtained to measure the scale of industrial transfer in each province, with a positive direction indicating an inbound industrial transfer and a negative direction indicating an outbound industrial transfer. 

#### 3.2.3. Control Variables

*Population size*. Because of the large differences in the administrative area and population size of each province, there is no comparability in terms of the absolute value of population size. Hence, Shao et al. [49] adopted a population density measurement, i.e., the ratio between the total population and administrative area of each province (unit: persons per km^2^) to measure the influence of provincial population agglomeration on local haze pollution.

*Level of economic development*. According to Kuznet’s hypothesis, the regional economic level has an impact on the local environmental quality. Therefore, this study included the economic development level as a variable. It was measured by per capita GDP (yuan/person) and street lightness intensity. From the availability of data, street lightness was measured by adopting the logarithm of the urban roadside lighting level provided by the Bureau of Statistics.

*Energy mix.* Fossil energy burning is the main cause of haze pollution, especially the burning of coal [50,51,52]. Because the main energy source in China is coal [53], energy mix is represented by the ratio of coal consumption to total energy consumption [54]. 

*Vehicle use*. The mileage of motor vehicles and local pollution levels are closely related. Vehicle exhaust emissions are not only a direct source of PM_10_, but also are the main source of secondary PM_10_. Some studies have reported that motor vehicle exhaust is the main source of PM_10_ in the urban atmosphere [55,56]. The ratio of highway mileage to the administrative area in each province was employed in this study to indicate the local vehicle use.

*Level of environmental regulation*. Environmental regulation by local government aims to alleviate the pressure of environmental pollution. According to some studies, intense environmental regulation has alleviated the pressure of local haze pollution. However, other studies have shown that the effect of environmental regulation on haze pollution is not satisfactory [57,58]. The promotion of official evaluation systems centered on gross national production has resulted in environmental regulatory behavior being imitated among provinces and regions, thus further degrading the environmental quality [59]. This study measured the intensity of local environmental regulation through a ratio of industrial pollution control investment and industrial added value in various regions.

*Industrial structure*. The degree of haze pollution can be enhanced by the development of secondary industrial sources, for example, exhaust emissions from burning fossil fuels and suspended dust caused by the development of the real estate industry. Industrial development consumes large amounts of energy, and industrial emissions directly increase the degree of haze pollution [60]. The industrial structure of each province was measured by the ratio of the industrial added value of each province to the gross national product.

Table 2 presents the definitions and abbreviations for these variables in the text.

*Meteorological conditions.* Meteorological conditions are also a significant factor affecting the degree of haze pollution. In 2017, the National People’s Congress proposed that great importance should be attached to the impact of relative humidity on haze pollution. In this study, the precipitation (unit: mm) and relative humidity of each region were used to measure local meteorological conditions.

#### 3.2.4. Sample Selection and Data Sources

Samples were collected for 30 provinces in China from 2008 to 2016. Tibet, Hong Kong, Macao, and Taiwan were excluded due to the availability of sample data. PM_10_ concentrations were obtained from the China Environmental Statistical Yearbook, raw industrial transfer data were collected from the China Industrial Statistical Yearbook, coal consumption and energy consumption (standard coal) data came from the China Energy Statistical Yearbook, and other data were sourced from the China Statistical Yearbook.

### 3.3. Descriptive Statistics of Variables

Descriptive statistics for the variables are provided in Table 3. There have been remarkable advances in the prevention and control of China’s haze pollution in recent years, although the average annual PM_10_ concentration remained seriously high at 101.11 μg/m^3^. The atmospheric quality of Hainan Province has been maintained well, with an annual PM_10_ concentration of only 34 μg/m^3^ at its lowest, but in 2013, the annual PM_10_ concentration in Hebei Province reached 305 μg/m^3^. To effectively alleviate the haze pollution situation, the “Ten Articles of Atmosphere” clearly identified that the elimination of outdated production methods will force industrial transformation and upgrades, and it is strictly prohibited to transfer outdated production methods and heavily polluting industry. This plan has achieved considerable results so far. The output value of China’s industrial manufacturing industry has shown an overall downward trend, while the output value of non-polluting industries has remained basically stable, but the output value of polluting industries has declined dramatically. Energy mix and industrial structure are the main factors influencing levels of haze pollution. China’s energy consumption structure is dominated by coal, with an average coal consumption ratio as high as 74.741%. The top three provinces in the country in terms of coal consumption are Shanxi, Inner Mongolia, and Guizhou, with an average coal consumption ratio of 91.99%. With the transformation and upgrading of industrial structure in China, the proportion of industrial production occurring in secondary industries in each province has presented the trend of a year-on-year decrease.

### 3.4. Framework of This Research

**Figure 1:**
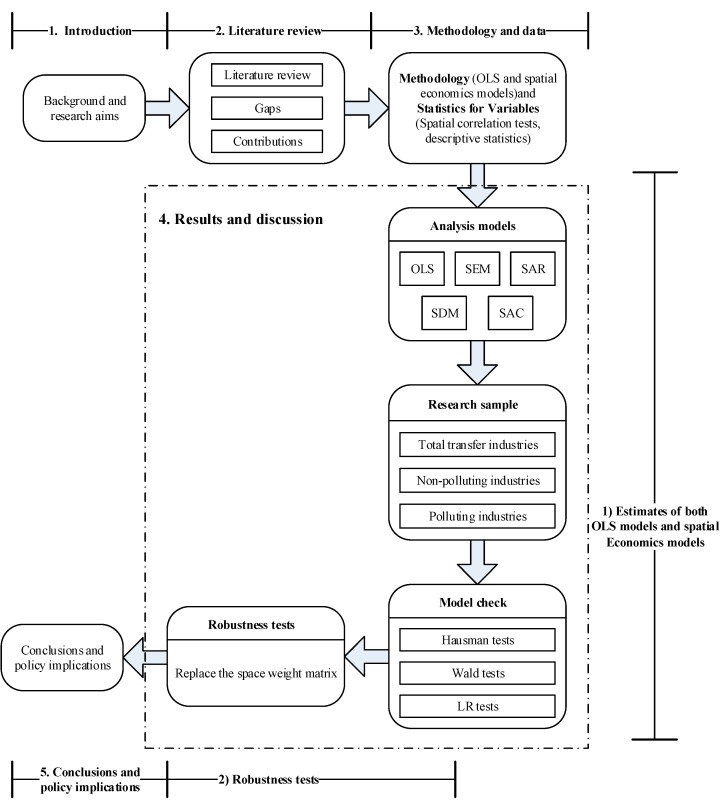
Framework of this research.

The overall framework of this study is presented in Figure 1. Based on the deficiency of previous literature, this research adopted the deviation-share method and the principal component analysis method to calculate the level and scale of industrial transfer in 30 provinces in China respectively. In particular, the traditional econometric model and space panel model were used in this study. Through Spatial Durbin Model, Spatial Auto-regression Model, Spatial Autocorrelation Model and Spatial Error Model, as well as Ordinary Least Square Model, the effect of industrial transfer on haze pollution is investigated. Taking into account the development gaps between different regions, China is divided into eastern, central and western regions to examine the environmental effect of industrial transfer. Next, the spatial weight matrix in the model was be replaced for analysis and the robustness of the benchmark model was tested. Finally, based on the empirical results, we summarized our research and put forward some policies and suggestions.

## 4. Results and Discussion

### 4.1. Variation Trend of Industrial Transfer and Haze Pollution

The “Environmental Air Quality Standard-GB 3075-2012” was promulgated and implemented in early 2016. It divides atmospheric environmental quality into two grades according to the annual PM_10_ concentration, and removes the third level standard. The first grade PM_10_ annual concentration limit is 40 μg/m^3^ and the second grade is 70 μg/m^3^. Due to the large time period considered in this study, the classification of the atmospheric environment used the previous Ambient Air Quality Standard-GB 3095-1996, which has an average annual first grade limit of 40 μg/m^3^, second grade limit of 100 μg/m^3^, and a third grade limit of 150 μg/m^3^.

**Figure 2:**
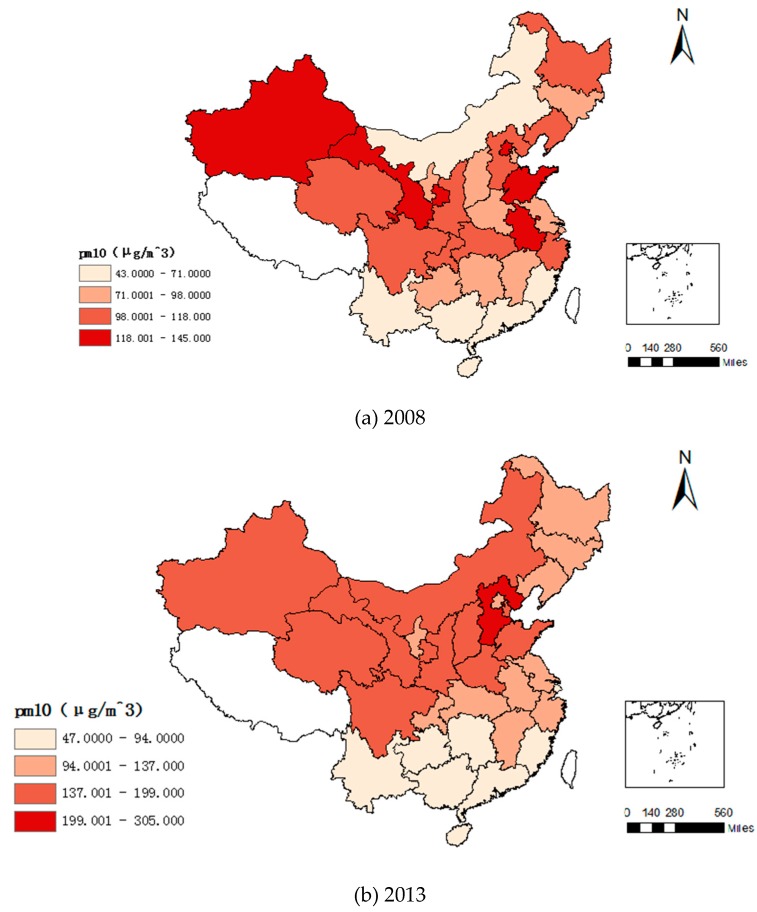

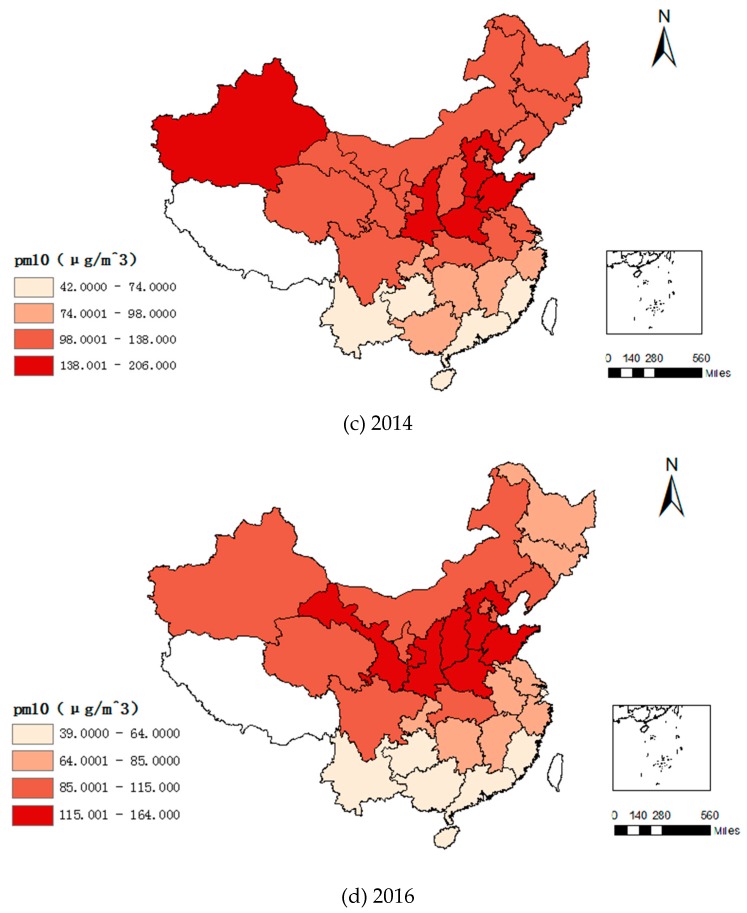
Variation trend of PM_10_ average concentration in each province: (**a**) 2008, (**b**) 2013, (**c**) 2014, (**d**) 2016.

As shown in Figure 2, among all the provinces and regions investigated in this study only Hainan Province had air quality at the first grade with respect to PM_10_. The implementation of the “Ten Articles of Atmosphere” has effectively alleviated haze pollution in China. In 2016, the PM_10_ concentration in Beijing was 16 μg/m^3^ lower than in 2013. Air quality in most of the central and eastern provinces, as represented by Hubei, Anhui, Jiangsu, Heilongjiang, Jilin, and Liaoning, has improved significantly, and the quality of the atmospheric environment has reached the second grade. Haze pollution in Qinghai, Gansu, Shaanxi, Henan, and Shandong has also been effectively alleviated. The quality of the atmospheric environment is stable within the limits of the third grade. As of 2016, the atmospheric environmental quality of Hebei Province still exceeded the third grade limit, but it had decreased by 46.2% compared to the average annual PM_10_ concentration in 2013. Although the reduction is remarkable, there is still a need for further prevention and control of haze pollution in Hebei Province.

**Figure 3:**
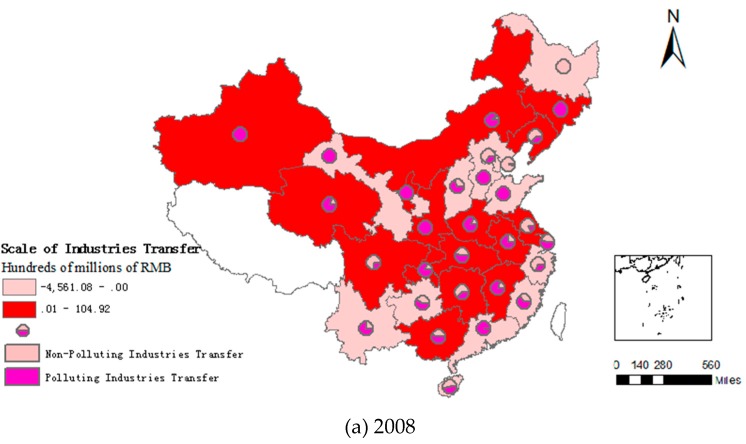

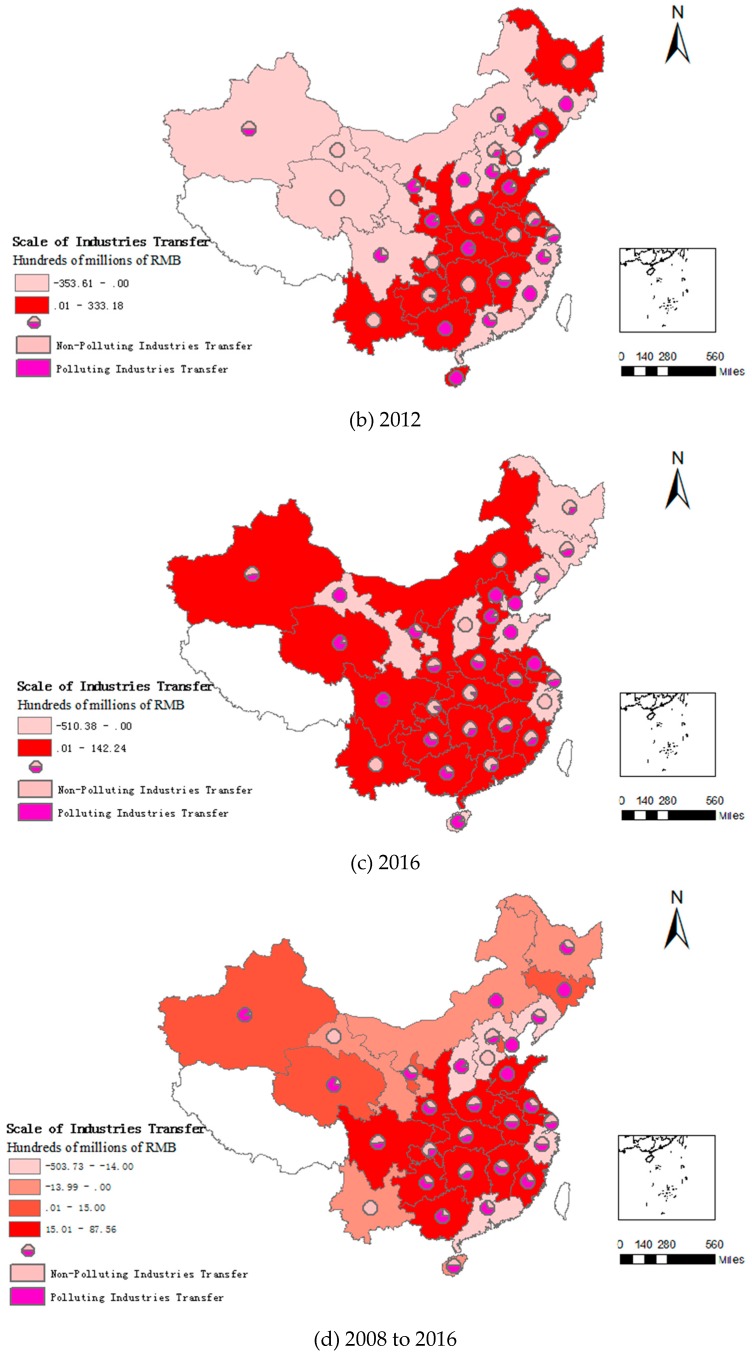
Spatial and temporal evolution of Chinese industry transfer: (**a**) 2008, (**b**) 2012, (**c**) 2016, (**d**) 2008 to 2016.

Figure 3 shows that China’s industrial manufacturing industry has displayed a trend to shift toward the central and western regions between 2008 and 2016. Compared with the western provinces, the central region has a high industrial transfer competitiveness, and is the main location for the transfer of industrial manufacturing from the eastern regions. Most of the central and western provinces have a relatively high proportion of polluting industries that could be transferred. For example, Xinjiang, Jilin, and Qinghai account for more than 80% of the transfer of polluting industries, with high-end industries still concentrated in the eastern region. The areas of industrial transfer are mainly concentrated in administrative districts such as Beijing, Shanghai, Hebei, Zhejiang, Liaoning, and Guangdong provinces, accounting for 95% of the total transferred volume. In addition to the eastern provinces, a small number of western provinces (e.g., Gansu, Inner Mongolia, Ningxia, and Heilongjiang) have also experienced small-scale outgoing industrial transfers.

### 4.2. Impact of Industrial Transfer on Haze Pollution

Based on the model settings described above, an OLS analysis of the impact of the overall industrial transfer status on the haze pollution of each province was conducted, with the results shown in Table 4. The spatial relativity of the model residuals was then analyzed.

**Figure 4:**
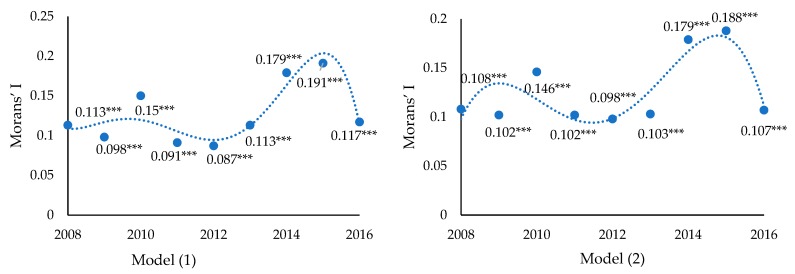
Identification for spatial correlation of OLS estimation error. Note: *** mean *p* < 0.01.

The regression results presented in Table 4 show that the overall industrial transfer level and scale have no significant effect on haze pollution. However, as shown in Figure 4, the OLS regression residuals has a significant spatial correlation, and therefore, the OLS analysis could not objectively display the relationship between variables. To improve the accuracy of the estimation, spatial panel measurements were employed in the analysis and the spatial correlations between provinces were taken into consideration. Prior to the spatial panel model regression, a spatial correlation test of the dependent variable *lnPM*_10_ was conducted, with the test results showing a significant spatial correlation (see Table 5). 

To explore the relationship between the overall industrial manufacturing transfer and haze pollution, the overall industrial transfer level and scale were adopted respectively. The SEM, SAR, SAC, and SDM were used in the analysis. A Hausman test was used to select the fixed effect spatial panel model (Taking the SDM as an example to explore the effect of industrial transfer on smog pollution, i.e., model (6), a Hausman test produced: chi2 (10) = 227.82, with a *p* value of 0.0000. When the effect of transfer scale on smog pollution was considered, i.e., model (10), a Hausman test produced: chi2(10) = 184.15, with a p value of 0.0000. The original hypothesis was rejected in both cases and the fixed effect model was selected.), with the results shown in Table 6.

Among models (3)–(10), a number of regression coefficients for SDM are significant in the fitting. A Wald test and LR were used to further test the fitting effect of the SDM and the original hypothesis was rejected after considering the test result (When exploring the impact of the overall industrial transfer level on smog pollution, the Wald test result is chi2 (10) = 38.86, with a p value of 0.0000; the L ratio test statistical result is chi2(10) = 45.23, with a p value of 0.0000. When exploring the impact of the overall industrial transfer scale on smog pollution, the Wald test result is chi2 (10) = 40.21, with a p value of 0.0000; the L ratio test statistical result is chi2 (10) = 43.63, with a p value of 0.0000.). When combined with the natural logarithmic values of each model, it is considered that the SDM has the best fitting effect, i.e., the influence of the two spatial conduction mechanisms of haze pollution contained in the SDM could not be ignored. Based on this, the SDM was selected for use in the analysis.

The results in Table 6 show that, whether measured by the industrial transfer level or the industrial transfer scale, the spatial lag coefficient is dramatically positive. This is further proof that the haze pollution in China has obvious spatial agglomeration characteristics. Taking the results of model (6) for example, every 1% increase in the PM_10_ concentration in a neighboring province will contribute to a 0.3% increase across the region. Hence prevention and control of haze should be a joint responsibility for the region. The leakage effect of haze pollution prevents unilateral haze governance from achieving the desired results. Models (6) and (10) produce different values of the independent coefficient, but the symbol and level of significance are basically consistent. The influence of various factors is analyzed and is explained in the following paragraphs:

*(1) Industrial transfer*. The level and scale of industrial transfer are both positively related to haze pollution. According to regional economic theory, industrial transfer not only promotes regional economic integration, but also alleviates the problem of central city resources and environmental pollution. Measured from either the level or scale of industrial transfer, the results of this study prove that the transfer of industrial manufacturing makes haze pollution more severe in areas where polluting industry is relocated too and to some extent alleviates the problem of industrial haze pollution in the original location. As the main region of outbound industrial transfer, the eastern developed areas have experienced an effective alleviation of haze pollution due to the large-scale transfer of industrial manufacturing. However, the transfer of industrial manufacturing will slow down the pace of local economic development. The transfer of secondary industry can effectively alleviate the dependence of local economic development on these industries, promoting the development of high tech industries, and forcing long term industrial transformation. This will not only optimize the industrial structure and create new economic growth points, but will also effectively alleviate regional environmental pressures.

Although haze pollution in areas where outbound industrial transfer is occurring can be effectively alleviated due to the transfer of industrial manufacturing, from the national perspective, industrial transfer cannot effectively control haze pollution, but would only enable its spatial redistribution. With the environmental cost incurred by the eastern cities increasing annually, some industrial manufacturing industries, especially polluting industries, have been actively shifting to the central and western regions through the implementation of policies and government interests. Central and western provinces are also actively promoting the transfer-in of eastern industries to provide opportunities for development. China has a vast territory, but there is a serious imbalance in the development of various regions. However, as China’s economy further develops, industrial transformation and the upgrading of various provinces and regions are only a matter of time. The central and western provinces have only considered economic development when undertaking industrial transfer. An overreliance on industrial manufacturing, especially polluting industries, will not achieve sustainable economic development. To alleviate the impact of polluting industries on the quality of the local atmospheric environment, the government should undertake a comprehensive consideration of the impact of enterprises on the regional economy and environment, encourage enterprises to research and develop technologies for green production, and also provide corresponding subsidies and preferential policies. 

*(2) Population density*. Population density has a significant effect on haze pollution. Demand for housing and transportation has increased as the population density has increased, which has directly and indirectly exacerbated haze pollution in the region. With an ever-increasing population density, local haze pollution can be alleviated by improving the efficiency of urban resource utilization and sharing facilities for pollution reduction. However, the positive externalities generated by population concentration have yet to be fully utilized, which is also an issue that the government should consider in future urban construction.

*(3) Level of economic development*. A negative correlation between the level of provincial economic development and haze pollution is identified, but the relationship is not significant whether measured from per capita GDP or lighting brightness (due to the possible multiple collinearity problem, when measuring the level of economic development in terms of per capita GDP and lighting brightness, a multi-collinearity test was conducted. The result is VIF = 2.89, indicating that there is no significant multicollinearity.). Based on data from 1998 to 2012, Shao et al. [45] discovered that the economic growth level and the haze pollution have a “U”-shaped relationship; some provinces have already exceeded the inflection point and arrived at the stage where the economic growth level is negatively related to haze pollution. Based on data from 2008 to 2016, it was found that the current level of economic development does not have an obvious effect on haze pollution; in contrast, it could alleviate the degree of local haze pollution. Although haze pollution is still persistent due to economic development, the results indicate that China has entered the stage where haze pollution and economic growth have become decoupled.

*(4) Energy mix*. Energy mix and haze pollution are positively correlated. Because China’s energy mix is dominated by coal [61], pollutants such as soot and SO_2_ that are released from coal burning are direct sources of haze. Coal accounts for as much as 70% of China’s energy consumption; however, the growth in coal consumption has increased haze pollution. To alleviate the impact of excessive coal consumption on haze pollution, the Chinese government should adopt policies such as actively guiding people to use green energy, and adjust the national energy consumption structure to move toward a green structure.

*(5) Vehicle use*. The local vehicle use is significantly positively correlated with the level of haze pollution. Provinces with convenient transportation links and a developed road system will have more motor vehicles and more exhaust gas emissions; hence, higher ${\mathrm{PM}}_{10}$ concentrations than provinces with poor transportation. Transportation is one of the main factors influencing haze pollution, and therefore the control of motor vehicle exhaust emissions would be an effective measure to alleviate local haze pollution. The construction of a convenient public transportation system and the widespread use of alternative energy vehicles would also be an effective means to alleviate haze pollution.

*(6) Level of environmental regulation*. Results from other spatial measurement models have shown that environmental regulation can significantly reduce haze pollution, but according to results gained from the SDM, environmental regulation can alleviate haze pollution levels, although the effect is not statistically significant. In this study, this variable was measured in various regions from the perspective of the implementation of environmental regulations, and it was found that such regulations do not fully meet their expectations. The investment output to alleviate industrial pollution is insufficient, and it is necessary to increase investment in industrial pollution control by the relevant government departments. Despite the insignificant effect of environmental regulations on haze pollution, the role of environmental regulation in the control of haze pollution cannot be ignored. Compared with remediation after the occurrence of haze pollution, it is better to prevent it from happening. All provinces and autonomous regions should formulate corresponding environmental protection regulations according to the local conditions, restrict the activity of enterprises that emit large amounts of dust and polluting gases, implement effective pollution gas emission standards, encourage cleaner production, enhance post-production supervision, appropriately raise the penalty for defaulting, and ensure the sustainable development of enterprises and regions.

*(7) Industrial structure*. The main factors causing haze pollution in China are the industrial structure, which is based on the secondary industry, and the extensive industrial development model. Some provinces still follow the development path of “pollution first and treatment later,” and the promotion of GDP is still a key factor that encourages local governments to ignore environmental problems and pursue economic benefits. The eastern region has recognized the disadvantages of pursuing economic development and neglecting environmental quality, and has shifted some industries to the central and western regions while eliminating outdated production methods. This has effectively alleviated haze pollution. From a global perspective, accelerating the upgrading of China’s industrial structure, supporting green industries, increasing the proportion of tertiary industry, and guiding the arrival of the post-industrial era in China are the main measures that will alleviate haze pollution.

*(8) Meteorological conditions*. Meteorological conditions in different provinces have different impacts on haze pollution. A high level of rainfall will decrease the PM_10_ concentration. Rainfall has a flushing effect on atmospheric pollution; thus, there are very few haze events in areas with high rainfall levels. However, in areas with relatively high levels of humidity, haze pollution is more serious [62]. In the fifth meeting of the 12th National Committee of the Chinese People’s Political Consultative Conference in 2017, the policy of “Attaching Great Importance to the Influence of Relative Humidity on Haze Pollution” was proposed, which indicates that high levels of relative humidity could accelerate the formation and transformation of haze.

Where there is a spatial spillover effect, changes in various influencing factors will not only affect local haze pollution, but also the level of haze pollution in neighboring provinces, causing a series of changes through feedbacks. The estimated coefficients of the SDM could directly reflect the influence of the independent variables on haze pollution. Usually the value of the direct effect is smaller than the estimated value of the coefficient. The influence of various influencing factors on haze pollution were decomposed into direct and indirect effects (i.e., spatial spillover effects) [37]. The direct effect refers to the total impact of a certain influencing factor on the local haze pollution level, including the spatial feedback effect. The indirect effect refers to the influence of a change in a local factor change on the level of haze pollution in the adjacent area. The results of the specific decomposition are presented in the Table 7.

As can be seen from the decomposition results, industrial transfer has a significant effect on haze pollution. Although the spatial spillover effect of industrial transfer is not statistically significant, its indirect effect accounts for more than 80% of the total effect. The effect of haze pollution in the region cannot be ignored. In addition, population density, energy mix, vehicle use, industrial structure, and relative humidity are important causes of haze pollution. The spatial spillover effect coefficient is positively related to population density and vehicle use, which means that through the spatial spillover effect, the population density and vehicle use in neighboring provinces will increase the level of haze pollution in a specific province. 

The level of economic development and precipitation amount can partially alleviate local haze pollution. The decomposition factors of economic development level, measured by per capita GDP and lighting brightness, indicate that the level of economic development directly mitigates the level of haze pollution in a specific region, although this effect is not significant and the coefficient is low. This indicates that the developed regions in China have gradually entered the stage where haze pollution and economic development are decoupled, which has been mainly achieved by transferring low-end industries to adjacent provinces while the high-end industries were retained and developed. The leakage of pollution from the industrial transfer allows the transferring provinces to continue their economic development, while alleviating local haze pollution. From a regional perspective, industries transferred out of developed regions can not only ease haze pollution, but also promote industrial transformation and upgrading. Although affecting the level of economic development in the short term, it will promote local economic development in the long run. However, from the national perspective, industrial relocation cannot eradicate haze pollution. If the main influencing factors are not controlled, industrial transfer becomes the last resort for alleviating haze pollution.

Environmental regulation has not played a direct role in suppressing haze pollution. The spatial spillover effect of environmental regulation may increase the level of haze pollution in neighboring provinces. This is because the expected environmental regulations are not realized due to imposition of environmental policies in neighboring provinces and concerns over the competitiveness of local industry.

### 4.3. Further Analysis

To further distinguish the impact of various types of industrial manufacturing transfer on haze pollution, and provide a basis for the provinces to undertake industrial transfer, 20 manufacturing industries were classified as either non-polluting or polluting industries, and the level and scale of industrial transfer was estimated using the OLS-[SAR\SEM]-SAC-SDM. The fixed effect model was selected in most cases, but the random effect model was used when the non-polluting industry transfer scale was used as the core explanatory variable, when the result of the Hausman test is chi2 (10) = 5.22 (*p* = 0.8760). When further testing the fitting effect of the SDM, following a Wald test and LR, the original hypothesis is rejected at a significance level of 1%. By combining the natural logarithmic values of the models, the SDM was considered to have the best fitting effect. The estimated SDM results are shown in Table 8.

The results shown in Table 6 and Table 8 show that the influence of control variables on haze pollution remained does not basically change. When analyzing the factors influencing industrial manufacturing transfer, it was found that the transfer of non-polluting industries would positively promote haze pollution, although this is not a significant influencing factor. However, the transfer of polluting industries would have a more significant effect on the local haze pollution level.

Table 9 shows the effect of the decomposition of the estimation results of models (11)–(14). Except for the core explanatory variables, the other influencing factors remain unchanged. The transfer of non-polluting industries into an area is not the main cause of local haze pollution. However, the transfer of polluting industries would not only cause significant pollution levels in the area where industry had relocated to, but there is also a dramatic positive correlation with the level of haze pollution in adjacent areas through the spatial spillover effect.

### 4.4. Robustness Test

The spatially weighted matrix used in our previous study is based on the gravity model, and this spatially weighted matrix based on the spatial geographic location was employed in a robustness test. Each element on the diagonal of the matrix is zero, and the values of the remaining elements are taken as *1/d_ij_,* where *d_ij_* represents the linear distance to the geographic center of the province in kilometers. Based on the results of a Hausman test the original hypothesis is significantly rejected by all models except models (17) and (18), and a fixed effect model was selected. According to the Wald test and LR results, and using the natural logarithmic values of each model, the SDM was considered to have the best fitting effect, with the model estimation results shown in Table 10.

After replacing the spatially weighted matrix, other than the economic development level, the estimation of all other variables is basically consistent, despite their being some differences in the coefficient sizes, and the significance and symbols are basically consistent with the original results. The results of the robustness test indicate that the level of economic development and the PM_10_ concentration are significantly negatively correlated; thus, verifying that provinces with a higher level of economic development had less haze pollution than other provinces. The eastern region has witnessed remarkable improvements in haze pollution as it has begun to decouple the level of economic development from haze pollution.

## 5. Conclusions

Under the gradual advancement of a strategy for industrial transfer, underdeveloped provinces actively undertake the transfer of industries from developed regions and seek economic development opportunities in the process, while developed regions have also realized industrial transformation and have begun upgrading their industry. Despite opportunities for economic development of the provinces provided by industrial transfer, the problem of pollution leakage during the process should not to be underestimated, because “Clear waters and lush mountains are invaluable assets.” To determine the impact of industrial transfer on haze pollution in China, this study used panel data for 30 provinces in China from 2008 to 2016. The shift-share method was employed to measure the transfer volume of each industrial manufacturing industry, and the entropy method was adopted to measure both the scale and level of industrial transfer of all industries, and non-polluting and polluting industries in each province. The relationships between haze levels and industrial transfer were analyzed by building a spatial measurement panel model based on the OLS-[SAR\SEM]-SAC-SDM concept.

It was found that industrial transfer will aggravate haze pollution in the receiving area, while the PM_10_ concentration in the transferring area will decrease. The transfer of non-polluting industry will not have a significant impact on haze pollution, but the transfer of polluting industry is positively related to haze pollution in not only the receiving area, but also adjacent areas through the spatial spillover effect. Therefore, underdeveloped regions’ government should objectively understand the impact of industrial transfer on the development of the region. While the effect of industrial transfer on the development of the receiving location is undeniable, the influence on local haze pollution should also not be underestimated, especially when polluting industry is transferred. Some developed provinces have alleviated local haze pollution by transferring their heavily polluting industries, but from a national perspective, this only treats the symptoms and not the root cause. For polluting industries, strengthening pollution control technology is the key to alleviating haze pollution. Therefore, when undertaking industrial transfer, the government should give preference to industries with low pollution levels, and provide subsidies and preferential policies to enterprises that research and develop pollution control technologies [63].

An assessment of the control variables shows that China has entered the stage where economic development and haze pollution are decoupled. However, there are significant positive spatial spillover effects of economic development factors, which would aggravate the degree of haze pollution in neighboring provinces. In view of the positive spatial correlation of haze pollution, haze treatment in a single province is less effective, and constructing a joint prevention and control mechanism in multiple provinces is the correct way to alleviate haze pollution. Efforts should be made to imitate the economic development cycle, construct a haze management system that operates across multiple provinces, and specifically to ensure the overall protection and control of the central and eastern regions where frequent haze pollution episodes occur. Regional governments should jointly formulate governance treaties, introduce multi-dimensional haze treatment programs, adjust and optimize the regional industrial structure, promote the use of clean energy, reduce coal consumption, and increase the incentives for the use of alternative energy vehicles.

As the main factor for promoting haze reduction, environmental regulation was not found to effectively alleviate haze pollution when its intensity of implementation was measured. Therefore, haze should be controlled from its source, and control measures have little effect when implemented after haze pollution occurs. Government department should formulate environmental protection laws from the prevention and control sources of haze pollution, and levy appropriate environmental taxes on the enterprises that are transferred. In addition, there is a need to force them to develop pollution control technologies, and enable the alleviation of haze pollution through market incentives, thereby achieving the synchronous development of economic development and ecological civilization construction.

## Figures and Tables

**Table 1:** Classification of 20 industrial manufacturing industries.

Classification	Number	Industry
Non-polluting industry	9	Processing of Food from Agricultural Products (1); Manufacture of Foods (2); Manufacture of Tobacco (3); Manufacture of Textile (4); Manufacture of General Purpose Machinery (5); Manufacture of Special Purpose Machinery (6); Manufacture of Railway, Ship, Aerospace and Other Transport Equipments (7); Manufacture of Computers, Communication and Other Electronic Equipment (8); Manufacture of Measuring Instruments and Machinery (9)
Pollution industry	11	Manufacture of Liquor, Beverages and Refined Tea (10); Manufacture of Paper and Paper Products (11); Processing of Petroleum, Coking and Processing of Nuclear Fuel (12); Manufacture of Raw Chemical Materials and Chemical Products (13); Manufacture of Chemical Fibres (14); Manufacture of Non-metallic Mineral Products (15); Smelting and Pressing of Ferrous Metals (16); Smelting and Pressing of Non-ferrous Metals (17); Manufacture of Metal Products (18); Manufacture of Medicines (19); Manufacture of Electrical Machinery and Apparatus (20)

**Table 2:** Definition of variables.

Variables	Symbol	Variable	Definition
Explanatory Variable	Y	PM_10_ Concentration	Annual PM_10_ Concentration in Provincial Cities
Core explanatory variable	IT	Industrial transfer level	Based on the shift-share method and entropy weight method, and the specific calculation steps are shown above
	SIT	Industrial transfer scale	
	IT_N_	Non-polluting Industrial transfer level	
	SIT_N_	Non-polluting industrial transfer Scale	
	IT_P_	Polluting Industrial transfer level	
	SIT_P_	Polluting industrial transfer Scale	
Control variable	POP	Population size.	Total population/administrative area
	RGDP	Per capita GDP	GDP/total population
	SL	Stable lighting brightness	The logarithm of city road lighting
	EM	Energy mix	Coal consumption/total energy consumption (standard coal)
	ROAD	Vehicle use	Highway mileage/administrative area
	ER	Level of environmental regulation	Industrial pollution control investment /industrial added value
	SEC	Industrial structure	Industrial added value/GDP
	RAIN	Rainfall capacity	Average annual rainfall
	HUMIDITY	Relative humidity	Absolute humidity/saturated absolute humidity

**Table 3:** Descriptive statistics for the variables.

Variables	Mean	Std. dev.	Min	Max
Y	101.11	31.99	34	305
IT	0.652	0.140	0.415	1.484
SIT	−17.111	290.037	−4561.08	333.177
IT_N_	0.639	0.148	0.405	1.431
SIT_N_	0.004	38.969	−257.151	147.557
IT_P_	0.662	0.150	0.423	1.817
SIT_P_	−17.115	281.681	−4552.76	285.445
POP	2791.789	1214.515	649	5967
RGDP	42675.15	22442.85	9855	118198
SL	13.159	0.742	11.086	15.068
EM	74.741	13.038	29.119	93.710
ROAD	13.814	23.167	0.083	125.397
ER	3.745	3.329	0.359	28.039
SEC	39.691	8.230	11.904	53.036
RAIN	950.842	569.967	148.8	2939.7
HUMIDITY	65.878	10.461	42	85

**Table 4:** OLS analysis estimation results.

Explanatory Variables	IT	SIT	POP	RGDP	SL	EM	ROAD	ER	SEC	RAIN	HUMIDITY	R−sq
Model (1)	0.193		0.104 ***	0.0231	0.0696 **	0.117	0.0215	−0.0227	0.174 **	−0.330 ***	−0.0453	0.510
	(1.56)		(2.83)	(0.59)	(2.47)	(1.32)	(1.37)	(−0.98)	(2.12)	(−8.07)	(−0.27)	
Model (2)		0.00702	0.100 ***	0.0205	0.0906 ***	0.0809	0.0256	−0.0229	0.181 **	−0.330 ***	−0.0778	0.510
		(1.57)	(2.73)	(0.52)	(3.58)	(0.91)	(1.61)	(−0.99)	(2.20)	(−8.08)	(−0.45)	

Note: *t* statistic values are shown in brackets; ** and *** mean *p* < 0.05 and 0.01 respectively.

**Table 5:** Global Morans’ I indicators for PM_10_ in 30 provinces in China during 2008–2016.

Year	Morans’I	E (I)	Sd (I)	z	*p*-Value
2008	0.079	−0.033	0.047	2.406	0.008
2009	0.151	−0.033	0.047	3.953	0.000
2010	0.128	−0.033	0.047	3.457	0.000
2011	0.109	−0.033	0.046	3.074	0.001
2012	0.114	−0.033	0.046	3.181	0.001
2013	0.226	−0.033	0.045	5.788	0.000
2014	0.289	−0.033	0.047	6.292	0.000
2015	0.259	−0.033	0.047	6.256	0.000
2016	0.303	−0.033	0.047	7.180	0.000

Note: E (I) is the expected value of I; E(I)=−1/(n−1); Sd (I) is the variance of I; z is the z test value of I; P is the probability.

**Table 6:** Regression results of the impact of overall industrial transfer on haze pollution.

Explanatory Variables	Overall Industrial Transfer Level				Overall Industrial Transfer Scale			
	SEM	SAR	SAC	SDM	SEM	SAR	SAC	SDM
	Model (3)	Model (4)	Model (5)	Model (6)	Model (7)	Model (8)	Model (9)	Model (10)
δ or λ	0.186	0.300 *	0.343 **	0.300 *	0.175	0.300 *	0.346 **	0.300 *
	(1.03)	(1.66)	(1.99)	(1.67)	(0.96)	(1.66)	(2.01)	(1.67)
IT	0.265 **	0.263 **	0.264 **	0.209 **				
	(2.26)	(2.21)	(2.26)	(2.09)				
SIT					0.00548	0.00562	0.00564	0.00612 *
					(1.34)	(1.36)	(1.39)	(1.85)
POP	0.073 **	0.0742 **	0.0745 **	0.181 ***	0.0647 **	0.0663 **	0.0666 **	0.189 ***
	(2.33)	(2.35)	(2.38)	(5.11)	(2.08)	(2.11)	(2.13)	(5.35)
RGDP	−0.0717	−0.0740	−0.0739	−0.0352	−0.0834	−0.0856	−0.0855	−0.0390
	(−1.29)	(−1.31)	(−1.34)	(−0.76)	(−1.49)	(−1.52)	(−1.54)	(−0.85)
SL	0.0497 *	0.0501 *	0.0496 *	−0.0386	0.0763 ***	0.0769 ***	0.0763 ***	−0.0105
	(1.91)	(1.92)	(1.86)	(−1.45)	(3.14)	(3.15)	(3.07)	(−0.41)
EM	0.0538	0.0512	0.0515	0.195**	0.0118	0.00971	0.00992	0.174*
	(0.53)	(0.50)	(0.51)	(2.13)	(0.12)	(0.09)	(0.10)	(1.93)
ROAD	0.0262 *	0.0266 *	0.0269 *	0.045 ***	0.0320 **	0.0322 **	0.0325 **	0.054 ***
	(1.87)	(1.90)	(1.93)	(3.26)	(2.29)	(2.33)	(2.35)	(4.07)
ER	−0.08 ***	−0.08 ***	−0.08 ***	−0.0193	−0.082 ***	−0.079 ***	−0.079 ***	−0.017
	(−3.70)	(−3.49)	(−3.52)	(−0.99)	(−3.66)	(−3.47)	(−3.50)	(−0.88)
SEC	0.219 ***	0.222 ***	0.224 ***	0.368 ***	0.242 ***	0.244 ***	0.245 ***	0.343 ***
	(2.71)	(2.73)	(2.73)	(3.99)	(3.01)	(3.00)	(3.01)	(3.72)
RAIN	−0.33 ***	−0.32 ***	−0.32 ***	−0.25 ***	−0.33 ***	−0.32 ***	−0.32 ***	−0.26 ***
	(−8.13)	(−7.80)	(−7.98)	(−6.09)	(−8.06)	(−7.74)	(−7.92)	(−6.27)
HUMIDITY	−0.171	−0.136	−0.139	0.654 ***	−0.201	−0.166	−0.169	0.622 ***
	(−1.06)	(−0.83)	(−0.87)	(4.21)	(−1.23)	(−1.01)	(−1.05)	(4.02)
W×T				0.697				
				(0.96)				
W×SIT								0.049 **
								(2.06)
R2	0.733	0.743	0.744	0.885	0.724	0.735	0.736	0.885
Log−L	53.127	55.539	55.466	119.059	51.489	53.986	53.903	120.063
AIC	−82.127	−87.078	−84.931	−194.12	−78.978	−83.972	−81.806	−196.126
BIC	−39.073	−43.897	−38.152	−114.95	−35.797	−40.791	−35.027	−116.961
Year	Yes	Yes	Yes	Yes	Yes	Yes	Yes	Yes

Note: *t* statistic values are shown in brackets; *, ** and *** mean *p* < 0.1, 0.05 and 0.01 respectively.

**Table 7:** Direct effect, space overflow effect and total effect of SDM model.

Explanatory Variables	Model (6)			Model (10)		
	Direct Effects	Indirect Effects	Total Effects	Direct Effects	Indirect Effects	Total Effects
IT	0.226 **	1.166	1.392			
	(2.04)	(0.84)	(0.95)			
SIT				0.00731 **	0.0774	0.0847
				(1.97)	(1.44)	(1.52)
POP	0.221 ***	2.371 *	2.592 *	0.232 ***	2.544 *	2.776 **
	(3.62)	(1.84)	(1.93)	(3.70)	(1.89)	(1.98)
RGDP	−0.00715	1.619 *	1.612	−0.0108	1.621 *	1.610
	(−0.13)	(1.70)	(1.63)	(−0.20)	(1.71)	(1.63)
SL	−0.0698 *	1.821 **	1.75 **	−0.0383	1.598 **	1.559 **
	(−1.73)	(2.38)	(2.37)	(−1.04)	(2.41)	(2.36)
EM	0.243 **	2.625	2.869	0.224 **	2.732	2.957
	(2.20)	(1.53)	(1.61)	(2.02)	(1.58)	(1.64)
ROAD	0.0555 ***	0.636 **	0.692 **	0.0656 ***	0.690 **	0.755 **
	(3.42)	(2.40)	(2.51)	(4.05)	(2.41)	(2.54)
ER	−0.0166	0.148	0.132	−0.0142	0.161	0.146
	(−0.80)	(0.72)	(0.62)	(−0.68)	(0.79)	(0.69)
SEC	0.396 ***	1.771	2.167 *	0.361 ***	1.186	1.547
	(3.72)	(1.60)	(1.83)	(3.46)	(1.15)	(1.40)
RAIN	−0.267 ***	−0.951 *	−1.22 **	−0.278 ***	−1.148 **	−1.43 **
	(−6.57)	(−1.86)	(−2.33)	(−6.62)	(−1.98)	(−2.39)
HUMIDITY	0.665 ***	0.370	1.035	0.635 ***	0.497	1.132
	(4.37)	(0.22)	(0.61)	(4.18)	(0.30)	(0.67)

Note: *t* statistic values are shown in brackets; *, ** and *** mean *p* < 0.1, 0.05 and 0.01 respectively.

**Table 8:** Regression results of the impact of different industrial transfer on haze pollution.

Explanatory Variables	Non−Polluting Industries Transfer		Polluting Industries Transfer	
	SDM	SDM	SDM	SDM
	Model (11)	Model (12)	Model (13)	Model (14)
δ or λ	0.300 *	0.626 ***	0.300	0.300 *
	(1.67)	(7.47)	(1.67)	(1.67)
IT_N_	0.143	/	/	/
	(1.60)			
SIT_N_	/	0.00376	/	/
		(1.02)		
IT_P_	/	/	0.200 *	/
			(2.15)	
SIT_P_	/	/	/	0.00998 ***
				(2.75)
POP	0.183 ***	0.169 ***	0.177 ***	0.200 ***
	(5.16)	(3.51)	(4.98)	(5.72)
RGDP	−0.0431	−0.101	−0.0354	−0.0245
	(−0.93)	(−1.14)	(−0.77)	(−0.55)
SL	−0.0335	0.00744	−0.0375	−0.00826
	(−1.27)	(0.17)	(−1.43)	(−0.33)
EM	0.203 **	0.220 **	0.181 *	0.172 *
	(2.21)	(2.06)	(1.99)	(1.93)
ROAD	0.0491 ***	0.0528 *	0.0421 **	0.0579 ***
	(3.62)	(1.81)	(3.01)	(4.24)
ER	−0.0226	0.00484	−0.0170	0.0248
	(−1.16)	(0.29)	(−0.87)	(0.86)
SEC	0.365 ***	0.175 *	0.380 ***	0.351 ***
	(3.97)	(1.93)	(4.14)	(3.89)
RAIN	−0.252 ***	−0.100 **	−0.248 ***	−0.252 ***
	(−6.06)	(−2.54)	(−6.01)	(−6.28)
HUMIDITY	0.643 ***	0.112	0.656 ***	0.594 ***
	(4.12)	(0.63)	(4.23)	(4.03)
W×IT_N_	0.231	/	/	/
	(0.34)			
W×SIT_N_	/	0.0687 *** (2.97)	/	/
W×IT_P_	/	/	0.887 (1.43)	/
W×SIT_P_	/	/	/	1.142 *** (3.07)
R2	0.8833	0.7052	0.8854	0.8883
Log−L	118.1496	156.4110	119.5896	123.0386
AIC	−195.1792	−264.8221	−192.2992	−202.0771
BIC	−116.014	−178.4599	−113.134	−122.9119
Year	Yes	Yes	Yes	Yes

Note: *t* statistic values are shown in brackets; *, ** and *** mean *p* < 0.1, 0.05 and 0.01 respectively.

**Table 9:** Decomposition results of the impact of different industrial transfer on haze pollution.

Explanatory Variables	Model (11)			Model (12)			Model (13)			Model (14)		
	Direct Effects	Indirect Effects	Total Effects	Direct Effects	Indirect Effects	Total Effects	Direct Effects	Indirect Effects	Total Effects	Direct Effects	Indirect Effects	Total Effects
IT_N_	0.147	0.411	0.558									
	(1.48)	(0.34)	(0.44)									
SIT_N_				0.00822	0.0826	0.0908						
				(1.37)	(1.32)	(1.39)						
IT_P_							0.222**	1.460	1.682			
							(2.18)	(1.14)	(1.26)			
SIT_P_										0.0115 ***	0.120 *	0.132 *
										(2.68)	(1.69)	(1.79)
POP	0.223 ***	2.420 *	2.644 *	0.233 ***	2.581 *	2.815 **	0.215 ***	2.312 *	2.528 *	0.242 ***	2.641 *	2.883 **
	(3.61)	(1.84)	(1.93)	(3.66)	(1.88)	(1.96)	(3.57)	(1.83)	(1.92)	(3.85)	(1.95)	(2.04)
RGDP	−0.0168	1.516	1.500	−0.00927	1.646 *	1.636	−0.00705	1.637*	1.630	−0.00171	1.811 *	1.813 *
	(−0.31)	(1.64)	(1.56)	(−0.17)	(1.69)	(1.62)	(−0.13)	(1.71)	(1.64)	(−0.03)	(1.81)	(1.75)
SL	−0.0635	1.75 **	1.69 **	−0.0358	1.62 **	1.58 **	−0.0692 *	1.86 **	1.79 **	−0.0366	1.57 **	1.533 **
	(−1.61)	(2.37)	(2.36)	(−0.96)	(2.40)	(2.35)	(−1.72)	(2.40)	(2.38)	(−1.02)	(2.42)	(2.37)
EM	0.253 **	2.714	2.967	0.223 **	2.551	2.774	0.229 **	2.586	2.815	0.263 **	3.207 *	3.470 *
	(2.27)	(1.56)	(1.63)	(2.02)	(1.50)	(1.56)	(2.08)	(1.52)	(1.59)	(2.34)	(1.74)	(1.80)
ROAD	0.0602 ***	0.666 **	0.726 **	0.0646 ***	0.673 **	0.737 **	0.0524 ***	0.613 **	0.665 **	0.0661 ***	0.678 **	0.745 **
	(3.72)	(2.42)	(2.54)	(4.00)	(2.40)	(2.53)	(3.21)	(2.38)	(2.48)	(4.16)	(2.42)	(2.56)
ER	−0.0200	0.144	0.124	−0.0139	0.197	0.183	−0.0143	0.151	0.137	−0.0138	0.142	0.128
	(−0.97)	(0.70)	(0.58)	(−0.67)	(0.94)	(0.84)	(−0.69)	(0.74)	(0.65)	(−0.68)	(0.71)	(0.61)
SEC	0.392 ***	1.733	2.125 *	0.353 ***	1.228	1.581	0.408 ***	1.824	2.232 *	0.328 ***	0.789	1.117
	(3.70)	(1.58)	(1.81)	(3.33)	(1.17)	(1.40)	(3.85)	(1.63)	(1.87)	(3.17)	(0.79)	(1.04)
RAIN	−0.267 ***	−0.913 *	−1.18 **	−0.280 ***	−1.081 *	−1.36 **	−0.263 ***	−0.963 *	−1.23 **	−0.281 ***	−1.28 **	−1.56 **
	(−6.55)	(−1.83)	(−2.31)	(−6.67)	(−1.93)	(−2.36)	(−6.50)	(−1.87)	(−2.33)	(−6.61)	(−2.08)	(−2.47)
HUMIDITY	0.650 ***	0.152	0.802	0.664 ***	0.435	1.099	0.668 ***	0.456	1.124	0.641 ***	0.833	1.474
	(4.26)	(0.09)	(0.48)	(4.37)	(0.26)	(0.65)	(4.41)	(0.27)	(0.66)	(4.25)	(0.50)	(0.86)

Note: *t* statistic values are shown in brackets; *, ** and *** mean *p* < 0.1, 0.05 and 0.01 respectively.

**Table 10:** Robust test results of spatial weight matrix based on geographic location.

Explanatory Variables	IT	SIT	IT_N_	SIT_N_	IT_P_	SIT_P_
	Model (15)	Model (16)	Model (17)	Model (18)	Model (19)	Model (20)
δ or λ	0.467 ***	0.462 ***	0.597 ***	0.607 ***	0.471 ***	0.457 ***
	(5.17)	(5.10)	(9.22)	(9.81)	(5.24)	(5.03)
IT	0.179 **					
	(1.98)					
SIT		0.00535 *				
		(1.73)				
IT_N_			0.0330			
			(0.44)			
SIT_N_				0.00107		
				(0.31)		
IT_P_					0.129 **	
					(2.64)	
SIT_P_						0.00735 **
						(2.12)
POP	0.0943 ***	0.0992 ***	0.116 ***	0.122 ***	0.0950 ***	0.102 ***
	(3.55)	(3.75)	(2.75)	(2.93)	(3.57)	(3.86)
RGDP	−0.116 ***	−0.116 ***	−0.125 ***	−0.130 ***	−0.123 ***	−0.116 ***
	(−2.66)	(−2.69)	(−2.50)	(−2.59)	(−2.83)	(−2.70)
SL	0.0365	0.0570	0.0690	0.0684	0.0396	0.0557
	(1.53)	(1.47)	(1.48)	(1.54)	(1.47)	(1.43)
EM	0.0368 **	0.00927 **	0.131	0.105	0.0438	0.0256
	(2.44)	(2.11)	(1.30)	(1.06)	(0.53)	(0.31)
ROAD	0.0241 *	0.0306 **	0.0211	0.0308	0.0277 **	0.0309 **
	(1.94)	(2.53)	(0.84)	(1.25)	(2.26)	(2.57)
ER	−0.0187	−0.0155	0.00454	0.0114	−0.0217	−0.0167
	(−1.04)	(−0.85)	(0.28)	(0.71)	(−1.20)	(−0.93)
SEC	0.343 ***	0.339 ***	0.194 **	0.206 ***	0.341 ***	0.333 ***
	(3.99)	(3.97)	(2.43)	(2.58)	(3.97)	(3.91)
RAIN	−0.223 ***	−0.218 ***	−0.082 **	−0.084 **	−0.219 ***	−0.214 ***
	(−5.64)	(−5.55)	(−2.09)	(−2.15)	(−5.53)	(−5.46)
HUMIDITY	0.711 ***	0.671 ***	0.168	0.137	0.696 ***	0.665 ***
	(4.54)	(4.30)	(0.93)	(0.76)	(4.42)	(4.27)
W×IT	0.321					
	(1.07)					
W×SIT		0.0140				
		(1.56)				
W×IT_N_			−0.158			
			(−0.92)			
W×SIT_N_				0.0213 ** (2.44)		
W×IT_P_					0.0538	
					(0.19)	
W×SIT_P_						0.0157
						(1.51)
R2	0.8875	0.8872	0.7403	0.7367	0.8841	0.8889
Log−L	130.0786	130.7456	158.9032	161.3626	129.0536	131.7788
AIC	−216.1571	−217.4912	−269.806	−274.725	−214.1072	−219.5577
BIC	−136.9918	−138.3259	−183.444	−188.363	−134.9419	−140.3924
Year	Yes	Yes	Yes	Yes	Yes	Yes

Note: *t* statistic values are shown in brackets; *, ** and *** mean *p* < 0.1, 0.05 and 0.01 respectively. The spatial interaction coefficient of control variables in SDM model is not reported.
